# A Pilot Study Examining Body Composition Classification Differences Between Body Mass Index and Bioelectrical Impedance Analysis in Children With High Levels of Physical Activity

**DOI:** 10.3389/fped.2021.724053

**Published:** 2021-11-15

**Authors:** David J. Farbo, Deborah J. Rhea

**Affiliations:** Department of Kinesiology, Texas Christian University, Fort Worth, TX, United States

**Keywords:** body mass index, bioelectrical impedance analysis, body fat, obesity, children, body composition

## Abstract

**Background:** Body mass index (BMI) is frequently labeled as “flawed” in assessing obesity since it cannot differentiate between muscle and fat leading to misclassifications of healthy individuals. Bioelectrical impedance analysis (BIA) may be a more accurate indicator of obesity since it can distinguish the difference between muscle and fat in children. This pilot study investigated discrepancies between BMI and BIA body composition classifications in children with high levels of physical activity.

**Methods:** Participants were selected from three elementary schools (*N* = 380, *K* = 76, 1st = 64, 2nd = 62, 3rd = 61, 4th = 83, and 5th = 34) receiving 60 min of outdoor, unstructured play daily. BIA scales were used to collect each child's body fat percentage and BMI score, then those numbers were categorized by BIA and BMI normative values as either underweight, healthy, overweight, or obese.

**Results:** Overall, 26% of the students were classified differently when using the normative classifications for BMI and BIA, with the largest discrepancy found in the overweight category at 38%. Similar inconsistencies were found when students were divided as younger (42%) vs older students (36%), and males (40%) vs. females (35%).

**Conclusions:** This pilot study demonstrated that there is a significant difference in how BMI and BIA discriminate between the different body composition categories. BIA consistently shows to be a more accurate tool in assessing obesity rates in children since it directly measures body fat.

## Introduction

Childhood obesity in the United States has steadily increased over the last 30 years and currently impacts over 13 million children nationwide (1). Research has shown that childhood obesity is an important risk factor for the development of Type 2 diabetes and cardiovascular diseases which can become chronic as body fat (BF) percentages increase (1). As a result of these types of diseases, ~150 billion dollars a year is spent on obesity related medical costs in the United States, with 14 billion dedicated to children (2).

Sedentary lifestyle choices are a significant contributor to the increased obesity rates and health disease rise seen in the United States today (3, 4). Sedentary behaviors are defined as any task that produces energy expenditure that is no greater than at rest, i.e., playing videogames from the couch (5). To prevent development of obesity, the Centers for Disease Control and Prevention (CDC) recommends that children between the ages of 6–17 engage in at least 60 min of moderate to vigorous physical activity (MVPA) daily (6). However, children today are shown to spend up to 8 h a day in sedentary activity, including the school environment. Consequently, only 24% of U.S. children participate in 60 min of physical activity (PA) daily (5, 6).

The loss of unstructured play and especially outdoors in the school setting is devastating for the healthy development of a child (3, 7). Physically, children refine gross and fine motor skills, coordination, muscular strength, and adaptability/balance (4, 8), while decreasing sedentary lifestyles, obesity risks, and health related chronic diseases (3, 5, 9). When children are given the opportunity to engage in unstructured play outside, some research has shown their PA patterns improve significantly more than when adults impose structured (PA) on them (4). Furthermore, other studies have shown that unstructured play will accumulate the recommended number of daily PA minutes through more moderate and vigorous activity than those in a structured environment (10).

Several research interventions have focused on promoting additional school PA opportunities with the goal of decreasing obesity trends in children (11–13). Limited obesity rate changes have been found in most elementary school focused interventions due to the short implementation intervals, i.e., 6–12 weeks, to assess the changes (11–13). A different type of school recess intervention called the LiiNK Project^®^ (Let's Inspire Innovation ‘N Kids), focuses on whole child development by implementing 60 min of outdoor, unstructured play throughout every day of a school year and a 15 min character development lesson daily to the school schedule. Prior to year 1 of the intervention, all teachers in grades pre-K through grade 1 from each school are trained to do the procedures associated with unstructured, outdoor play breaks and the character curriculum. In the last half of year 1 implementation, grade 2 teachers are trained to do the procedures, then grade 3 teachers are trained in the last half of year 2. Implementation always begins the fall after spring training. The school can choose to advance the project into grades 4 and 5 during the 3rd year of the project. The project requires all teachers per grade level to take their students outside simultaneously four times daily for 15 min each time and they cannot withhold recess for tutoring or punishment.

Previous LiiNK Project studies have shown many whole child benefits that include improved on task classroom behavior (14), attentional focus (15), and positive emotional states (16, 17). Accelerometer results have shown LiiNK students take ~8,700 steps and achieve 140 min in MVPA each day while they are in school (16, 18). Other studies have reported that when children aged 7–11 engage in 55–66 min of MVPA and ~10,000 steps per day, it lowers their chances of developing excess BF and obesity related health risks (19, 20). However, these statistics were not seen using BMI as the assessment tool to classify students who are overweight or obese with LiiNK students. Only 7% of the LiiNK students who were classified as overweight or obese at the start of the intervention shifted to the healthy category after a 3 year period (21). One reason for these inconclusive results may be due to the use of BMI to assess overweight/obesity rates in these children with a measure that may not accurately assess BF with higher levels of PA throughout the day.

Much attention has been placed on assessing whether a person is considered healthy or unhealthy with weight or BF. Several terms have been used to guide the public's understanding of what target numbers should be. Body composition has been used as a general term and encompasses whole body weight components consisting of fluid, muscle, bone, organs, skin tissue, and fat (22). We can then classify individuals into different categories based on body composition that include underweight, healthy, overweight, or obese. The category related to most health concerns for the general public is obesity which is defined as excessive BF accumulation that presents an increased risk for morbidity and mortality (22, 23). BMI has been utilized for many years as an estimate of body composition for its simplicity and correlation with fat accumulation and health risks in obese individuals (24). BMI is a height/weight ratio score that uses CDC provided normative charts to categorize each student by age and gender as either underweight, healthy, overweight, or obese (1). The use of BMI as a determiner of BF has been scrutinized over the years because it uses an estimation of overall BF to categorize individuals instead of measuring BF directly, which can sometimes result in misclassification of those who are not overweight/obese (22, 25). For example, children who have BMI scores one or two points above the percentile cut-offs for overweight or obese may not actually have high levels of fat. Rather, they may have a higher weight due to the development of more muscle mass, which means they do not fall into the overweight or obese category as BMI would suggest. This chance of error to misclassify individuals may help explain why prior intervention studies using BMI scores as the indicator of obesity have not produced conclusive results as some of these children may have been categorized as overweight or obese when they were not. For children who may have a higher amount of muscle mass due to higher levels of MVPA, using a more accurate BF assessment is needed to properly assess obesity trends (25).

Many techniques are used to collect body composition such as densitometry, BIA, dual energy X-ray absorptiometry (DXA), waist circumference, and skinfolds (26). Densitometry and DXA are typically known as the gold standard for measuring BF. However, they are both unrealistic options for most researchers as they are expensive, labor intensive, and immobile for large scale studies out in the field (27). Waist circumference is similar to BMI in that it only gives an estimate of BF and still has a chance to misclassify children (28). Skinfolds accuracy depends on whether the individual who is administering the skinfolds has sufficient training and follows the standardized protocols for the measurements (26). Additionally, skinfolds can be time consuming and collecting data on a large number of participants can be daunting (26).

BIA is able to determine an estimate of fat mass, fat free mass, water weight, and bone density that shows a moderate to strong association with results provided by DXA (26). These measurements are determined by use of an unnoticeable electrical current that is sent throughout the body by either hand to foot or foot to foot metal plates (26, 29). This assessment has been recommended as an alternative BF measure in fitness manuals such as Fitnessgram to collect children's data in a school setting due to reducing human error, convenience of using it in large group settings, and BF percentage accuracy across different populations (26, 27, 30). Additionally, most BIA scales are durable, easily transportable, and only require monthly recalibration so they provide reliable results without many additional resources or costs (27, 29). However, accuracy can depend on the choice of device, hydration level (26), and maturation level of the participant (29). Even with these limitations, BIA still has high sensitivity and specificity in classifying individuals into different body composition categories based on BF, which may make it a more reliable measure of obesity than BMI.

Therefore, the purpose of this pilot study was to determine if there was a difference in how BMI and BIA classified students into the four body composition categories. Since this was the first time the LiiNK researchers had used the BIA assessment tool and were trying to determine BIA and BMI category accuracy, a convenience sample of participants were assessed from different grade levels and schools participating in the LiiNK Project. It was hypothesized that BIA measurements would be positively correlated with BMI considering that BMI is known to have a moderate association with high levels of BF in obese individuals. A second hypothesis was BMI and BIA would classify students into body composition categories differently. Finally, it was hypothesized that the body composition classifications would be different between the two measurements by age and gender also.

## Methods

### Participants

This pilot study used a one group posttest only design and participants were selected using a convenience sample from three North Texas public elementary schools participating in the LiiNK Project intervention. This intervention was approved through a partnership between the school district and the University research team to implement and measure aspects of whole child development. All participants had been participating in four unstructured recesses daily for the past 3–5 years. All students were included who received parental consent and followed the normal school schedule. Students were excluded if they wore a pacemaker, had an injury that prevented them from participating in recess regularly or from being able to stand on the scale with their shoes off. Since this was a pilot study that utilized a convenience sample of students who received parental consent, power estimations were not calculated before recruitment. Table 1 provides the total number of participants (*N* = 380) by school, age, and gender. The ethnicity of students at the schools selected represented 40% White, 40% Hispanic, 15% Black, and 5% other. Since this study sought to examine differences between BMI and BIA by age and gender, ethnicity was not considered in recruitment of participants or in data analysis.

**Table 1:** Participants by grade and gender.

**Grade**	**Gender**	***N***
K	M	39 (10%)
	F	37 (10%)
1st	M	36 (10%)
	F	28 (7%)
2nd	M	39 (10%)
	F	23 (6%)
3rd	M	34 (9%)
	F	27 (7%)
4th	M	46 (12%)
	F	37 (10%)
5th	M	19 (5%)
	F	15 (4%)
**Total**		380 (100%)

### Measures

#### Body Composition

Bioelectrical impedance analysis was used to measure body composition among participants. The BIA device used was the Tanita^®^ BF 2000, which is proven to be valid and reliable in measuring BF and fat-free mass among elementary aged children (31). The scale sends a small, unnoticeable electrical signal throughout the body starting at the feet *via* metal plates located at the base of the device. Fat is a poor conductor of electricity due to a low water content and will cause resistance on the electrical current. The greater impedance present in the current, the higher percentage of BF the scale will calculate. The height of the participant is measured and entered into the software program beforehand for more accurate body composition measurements. Once height is entered, participants stand on the metal plates with their shoes off for about 10–15 s and the scale will calculate fat mass, fat free mass, BF percentage, and BMI.

Results were kept confidential from participants since the scales do not contain a screen and all data is uploaded to a computer *via* Bluetooth. This feature is important when working with a young population as researchers do not want to initiate any negative psychological effects as a result of children seeing their results. The software program associated with the BIA scale categorizes each student into underweight, healthy, overweight, or obese based on their BF percentage/BMI score, age, and gender. The BMI reference curve scores used to categorize males and females are provided by the CDC (1). McCarthy et al. (32) provide the normative values for BF percentages for each gender. For example, for a 9 year old male, a BMI score below the 5th percentile (~14) would classify him as underweight, a score between the 5th and 85th percentile (~14–~18.6) would be healthy a score between the 85th and 95th percentile (~18.6–~21) would be overweight, and a score above the 95th percentile (>21) would be obese. For that same 9 year old male, the BF normative values would reflect the underweight category to be <14% BF, healthy would be 14–22% BF, overweight would be 22–27% BF, and obese would be >27% BF.

### Procedures

The University Institutional Review Board approved the cross-sectional pilot study design. Three schools from the same district participating in the LiiNK intervention were chosen to be assessed with the BIA scales. Comparison schools were not assessed for this pilot since we wanted to identify differences between two body composition tools first for accuracy prior to engaging a different school setting. Once the researchers selected which schools would be assessed, principals were notified about procedures for the study and approval was given prior to the collection of BIA data. Parents were provided and returned informed consent letters to either approve or deny their child's participation in the BIA study and only students that received parental consent were able to participate. Students were able to decline participation at any time if they did not feel comfortable getting their measurements taken using the BIA scale.

Physical education (PE) teachers were asked to provide class rosters, height, and date of birth one week before data collection on an Excel template that was provided by the lead researcher for all children approved to participate. This data was entered into the BIA software prior to school arrival and each student was given a unique ID number to track their data. On the collection day, the researcher arrived at the school ~20 min before the start of the PE class to set up the BIA scale station.

When the PE class started, the physical education teacher sent groups of 10 students in alphabetical order to a corner of the gym where the researcher and BIA scale were located. Students were asked if they wanted to participate in this assessment by standing on the scale with their shoes and socks off. If they consented, then they were instructed to remove their socks and shoes and when their names were called, they would stand on the metal plates located at the bottom of the scale. After about 15–20 s, the scale flashed a green light signifying that the measurement was complete, and students returned to the class activity. The scale was then disinfected with alcohol wipes, the next student was called to stand on the scale, and the process was repeated. Once that group was measured, the teacher would call over the next group of 10 students to the scale station to repeat the same procedures. Each class took ~30 min to be assessed.

At the end of collection for the day, all data was downloaded from the computer software program into Excel. Each daily Excel sheet was then added to the master data sheet from other days of data collection. Two schools required 2 days of data collection while the final school only required 1 day of collection for a total of five data collection days over 2 weeks. The difference in collection days was due to two schools alternating days in which students attended their PE class while the other school saw all children from each grade level daily. Ideally, students should be measured in a fasted state and well-hydrated when using BIA to produce the most accurate results. However, with such a wide variety of schedules and needs of the students, this was not possible for this study. As a result, data was collected at all schools in the morning and afternoon.

### Data Analysis

All data was analyzed using IBM SPSS statistics version 25. Descriptive statistics were used to distinguish the characteristics of the group as a whole, by grade, and by gender. The first hypothesis was tested using Pearson's correlation coefficient to test the relationship between BMI and BIA before moving forward with further analyses. To test the second and third hypotheses, students were first categorized using CDC's BMI normative age and gender values labeled as underweight, healthy, overweight, and obese (1). The students were then categorized using McCarthy et al. (32) normative values for BF percentage after using the BIA scale to classify them as underweight, healthy, overweight, and obese. The second and third hypotheses were tested using non parametric chi square tests to examine classification differences between BMI and BIA. For the third hypothesis, younger children were identified as those in Kindergarten, first, and second grades (*N* = 202) and older children were identified as those in third, fourth, and fifth grades (*N* = 178).

## Results

### BMI/BIA Descriptive Statistics

Since this was a pilot study and students were only measured at one time point, no attrition was experienced in the current sample (*N* = 380). Table 2 shows BMI/BIA means and standard deviations by grade and gender. Fifth grade numbers are noticeably lower than other grade levels due to only one school having the LiiNK intervention through fifth grade at the time of collection. Overall, older students recorded higher BMI and BF percentages than younger students. In addition, females recorded higher BMI, BF percentages, and fat mass than males while males showed higher fat free mass than females.

**Table 2:** Descriptive statistics-means and standard deviations.

	**Weight (Kg)**	**Height (m)**	**BMI**	**Body fat (%)**	**Fat mass (Kg)**	**Fat free mass (Kg)**
	**mean (SD)**	**mean (SD)**			**mean (SD)**	**mean (SD)**
**Gender**						
Male	29.77 (8.99)	1.31 (0.11)	17.12 (3.33)	20.03 (7.22)	6.35 (4.09)	23.43 (5.89)
Female	29.88 (11.22)	1.30 (0.12)	17.25 (4.00)	21.68 (8.67)	7.23 (5.77)	22.66 (6.12)
**Grade**						
Kindergarten	21.47 (4.98)	1.16 (0.06)	15.85 (2.85)	20.17 (7.01)	4,64 (2.87)	16.84 (2.38)
1st	24.71 (5.23)	1.24 (0.06)	16.06 (3.17)	19.22 (7.64)	5.08 (3.30)	19.63 (2.59)
2nd	28.64 (6.45)	1.30 (0.06)	16.96 (3.20)	21.36 (7.70)	6.53 (4.06)	22.12 (3.06)
3rd	30.81 (8.97)	1.32 (0.07)	17.38 (4.04)	20.99 (8.32)	7.10 (5.27)	23.72 (4.25)
4th	36.18 (9.03)	1.40 (0.07)	18.27 (3.52)	21.29 (7.97)	8.30 (5.16)	27.89 (4.49)
5th	53.86 (11.08)	1.47 (0.05)	19.63 (4.32)	22.14 (9.77)	10.41 (7.42)	32.52 (4.74)
Total	29.82 (10.01)	1.30 (0.12)	17.18 (3.63)	20.76 (7.92)	6.73 (4.92)	23.09 (6.00)

### Hypothesis 1: BMI and BIA Correlation

The Pearson Product correlation coefficient was used to examine the relationship between the BMI and BIA. The results revealed that there was a strong positive correlation between BMI and BIA, *r* = 0.85, *p* < 0.001. These results suggest that what is measured with BMI is very similar to what is measured with BIA. This confirmation of measurement allows for further analyses of the differences in accuracy between BMI and BIA.

### Hypothesis 2: BIA vs. BMI Differences

The chi-square test results revealed a significant difference between how BMI classified students and how BIA classified those same students, $x_{\left(9\right)}^{2}$ = 470.51, *p* < 0.001. Table 3 details where the exact differences between the two measurements occurred. According to BMI, 40 students were underweight, 221 students were healthy, 52 were overweight, and 67 were obese, which is presented in the BMI total column. The BIA columns provide information on how the BMI total in each row were categorized according to BIA. For example, of the 40 students classified as underweight according to BMI, 26 were classified as underweight and 14 were classified as healthy according to BIA. This means that 14 of the 40 students were categorized differently between the two measurements, resulting in a 35% difference for the underweight category. These results support the second hypothesis that a classification difference was found between BMI and BIA. Further support is three of the four categories had a 28–38% discrepancy between the two assessment tools. The only low percentage discrepancy was the obese category (6%).

**Table 3:** Classification differences between BMI and BIA by all students.

		**BIA**				
**BMI total**	**BMI classification**	**Underweight**	**Healthy**	**Overweight**	**Obese**	**% Difference**
40	Underweight	26	14	0	0	35%
221	Healthy	37	160	23	1	28%
52	Overweight	0	10	32	10	38%
67	Obese	0	0	4	63	6%

### Hypothesis 3: BMI and BIA Differences by Age and Gender

Four chi-square tests were used to test the third hypothesis which stated there would be classification differences between BMI and BIA based on age and gender. For the age analysis, students were divided into two groups, younger (*N* = 202) and older (*N* = 178). Chi-square analysis revealed there was a significant difference between how BMI classified students and how BIA classified those same students for the younger age group, $x_{\left(9\right)}^{2}$ = 263.71, *p* < 0.001, and the older age group, $x_{\left(9\right)}^{2}$ = 225.32, *p* < 0.001. For gender, chi-square analysis also revealed a significant difference between how BMI classified students and how BIA classified those same students for both males, $x_{\left(9\right)}^{2}$ = 223.75, *p* < 0.001, and females, $x_{\left(9\right)}^{2}$ = 267.57, *p* < 0.001. The assumption for running chi-square states that there should be no cells in the contingency table with an expected value less than five. In these results, males and the younger age group reasonably met this assumption, whereas for females and the older age group they were not satisfactory. Therefore, caution should be taken when interpreting the female and older age group results. Table 4 provides where exact differences between the two measurements occurred for each test. The underweight, healthy, and overweight categories had fairly large discrepancies for the younger children and the older children. The overweight category had the largest discrepancy for males and females, while males had a much larger discrepancy than females in the underweight category. All categories had discrepancies, but the largest discrepancies across the participants was with underweight, healthy, and overweight categories.

**Table 4:** Classification differences between BMI and BIA by age and gender.

**BMI**		**BIA**				
**BMI total**	**BMI category**	**Underweight**	**Healthy**	**Overweight**	**Obese**	**% Difference**
**Younger children**						
31	Underweight	19	12	0	0	39%
115	Healthy	11	86	17	1	25%
24	Overweight	0	3	14	7	42%
32	Obese	0	0	0	32	0%
**Older children**						
9	Underweight	7	2	0	0	22%
106	Healthy	26	74	6	0	30%
28	Overweight	0	7	18	3	36%
35	Obese	0	0	4	31	11%
**Males**						
23	Underweight	11	12	0	0	52%
117	Healthy	17	79	20	1	32%
35	Overweight	0	7	21	7	40%
38	Obese	0	0	3	35	8%
**Females**						
17	Underweight	15	2	0	0	12%
104	Healthy	20	81	3	0	22%
17	Overweight	0	3	11	3	35%
29	Obese	0	0	1	28	3%

## Discussion

This pilot study sought to examine body composition classification differences between two measures of body composition in elementary school children with 60 min of active play daily. BMI has been used for many years since it is fairly easy to collect and demonstrates a moderate correlation in assessing BF in obese individuals (24). The problem with BMI is that it only provides an estimate of body composition that does not truly distinguish a difference between BF and muscle mass, which can lead to the misclassification of healthy individuals (25). This is especially true when the population measured has a high amount of MVPA as they may have a high weight due to more muscle mass than an accurate BF percentage. Previous studies suggest that 55–66 min of MVPA and ~10,000 steps per day in children aged 7–11 will lower their odds of developing excess BF and obesity related health risks (19, 20). LiiNK Project children, measured with accelerometers over 2 weeks for the 7.5 h school day, exceed these recommendations by taking ~9,000 steps and engaging in ~140 min of MVPA per day during the school hours only (18), which should result in lower obesity rates in LiiNK Project schools. However, prior LiiNK data only demonstrates marginal changes in overweight and obesity classifications over a 3 year period when BMI is used to measure obesity rates (21). Since these children may be experiencing an increase in muscle mass as a result of higher levels of MVPA, BIA seems to show a more accurate way to determine obesity rates since it is able to distinguish the difference between muscle mass and BF (26).

The results reveal many instances in which there was a significant difference in how each measure classified children. Overall, on average, there was a 26% difference between the two measures. BMI classified 99 of the 380 students differently than BIA in all categories, with the overweight category showing the biggest difference at 38%. Similar differences were also reflected in the overweight category by age. Younger children reflected a 42% discrepancy in the overweight category between BMI and BIA, while there was a 36% discrepancy in the overweight category for the older children between the two measurements. This is consistent with Alves et al. (33), who found a 45% difference between BMI and BIA in classifying children aged 7–10 years old who were in the overweight category. Freedman and Sherry (34) also discovered that 26% of their participants aged 6–18 were classified as overweight or obese by BMI when they actually had healthy levels of BF. Similar differences can be seen in the overweight classification for both males (40%) and females (35%). This is similar to Alves et al. (33), who found a 50% difference in males and 37% difference in females between the two measures in the overweight classification. It is clear from these findings that BMI is inaccurate in classifying individuals who are on the border line of either healthy or overweight. These studies support the hypothesis that past LiiNK students who were 1 or 2 points higher than the BMI cutoff for overweight were being misclassified and actually should be classified as healthy.

The smallest difference between the two measures across all students was in the obese classification with only a 6% discrepancy. Other research has shown that BMI is accurate in determining obesity in individuals with high levels of fat, or those that are severely obese, which is consistent with the results of this study (35). This relationship seems to be consistent when examining younger children and females as they had the smallest difference in the obese classification than any other sub groups at 0 and 3%. In general, there appears to be much more disagreement in the overweight and obese categories in older children and males when compared to younger children and females. These results are similar to the findings of Vanderwall et al. (36) who found that BMI is a poor predictor of BF percentage in children 9 years old and younger and a moderate predictor in children between the ages of 9–18. This error in students under 9 years old supports the hypothesis that BMI was not a valid tool to assess overweight or obesity rates of students in the LiiNK project as past data has mainly examined trends of students in grades K-2 (5–8 years old) (21). Nwizu et al. (37) also found that the accuracy of BMI to predict BF percentage varies between male and female adolescents as 46% of their male participants with a healthy BF percentage were classified as overweight or obese by BMI compared to only 26% of females. They propose that these differences are a result of a higher accumulation of muscle mass in male participants, which is what could be happening with the males in the current study as a result of the increased time for PA daily (37).

Another interesting trend was in the underweight classification, where BMI demonstrated a moderately high discrepancy across all ages and gender. Houska et al. (38) also found that BMI was inconsistent in classifying underweight collegiate female athletes and the authors concluded that BMI is not a useful tool when the population being measured may have more lean mass than BF. This could support the claim that students actively playing at least 60 min daily are being misclassified due to higher muscle mass (35). Since most of the students selected in this study were entering their fourth or 5th year of the intervention, they could be experiencing longitudinal increases in muscle mass as they age, leading to misclassifications when using BMI. Although the results presented here show that there is a difference between the two measurements, BMI is still frequently used in research studies that aim to decrease obesity rates in children by increasing PA (11–13). These studies report little to no changes in obesity rates at the end of a PA intervention, but their results may be questionable since they used BMI and they may discover different finding if they used BIA. Researchers who continue to use BMI as an assessment of body composition in children who have higher amounts of PA are at risk of producing inaccurate results. Some may not use BIA due to lack of awareness that another assessment tool is available or lack of funds to purchase equipment (26, 29). However, BIA continues to reflect a more accurate assessment of obesity since these results are consistent with at least four other studies that have compared BMI and BIA classification differences (33, 34, 36, 37). BIA also measures BF directly which is a better indicator of overall health than a BMI score. This could be especially important for those children who are one or two points above or below the percentile cutoffs according to BMI. The use of BIA may then lead to more definitive results in future active play intervention studies focused on obesity rate determination in children.

### Limitations

The first limitation of this study is the sample size when age and gender are assessed categorically with chi-square tests. The results showed that the assumptions of chi-square were not supported for females and older age children. A larger sample size would provide better insight about the differences in females and older children. The second limitation of this study was the variability in the hydration level and time of day BIA measurements were collected. Ideally, BIA measurements should be measured when the participant is fully hydrated, and before eating or exercise has taken place. However, it was nearly impossible to measure all students at a time in which they were fully hydrated, had not eaten, or engaged in PA before-hand due to the nature of the LiiNK intervention. These factors could have produced inaccurate assessments of BF percentage which would lead to a misclassification, but may be more unlikely since they match so well with other studies examining differences in these measurements (33, 34, 36, 37). Another limitation is ethnic differences that may have contributed to the extent and direction of the error seen between the two measures. Different ethnicities may experience higher variability in bone density, limb length, and even maturation rate, all of which could have led to a misclassification when using BF produced by BIA since the scale cannot account for these differences. However, analyses by ethnicity were not possible for the current study due to a smaller number of diverse groups. Finally, a COVID-19 limitation is the inability to compare BIA results to a gold standard body composition measure such as DXA to calculate specificity and sensitivity of the scale used in the current study. Other studies have confirmed the scale to be valid and reliable when compared to DXA, however the population used in the current study experienced higher levels of PA than normal which may alter the sensitivity and specificity of the scales. Confirming these results in future studies would strengthen the accuracy of BIA in assessing obesity rates in children with higher levels of PA.

### Future Directions

Future studies should consider a larger sample size since this was only a pilot study that utilized a convenience sample of participants. This is especially the case when examining any differences between the measures in females and older children in the current study, which had a limited sample size and were not satisfactory to meet the assumptions of the chi-square analyses. Future research should examine specificity and sensitivity for the BIA scale used in the current study to confirm its accuracy in determining obesity rates in children with high levels of PA. Additionally, future research among this population should examine differences in BF percentage between children in the LiiNK project and a control school with <20 min of recess daily. It would also be beneficial to examine race and ethnic differences in BF percentage between LiiNK and control school students since each can have an effect on body composition. An examination of the longitudinal effects of the LiiNK project on BF percentage would also be appropriate for future research. LiiNK project students may be experiencing greater increases in muscle mass and decreases in BF than control school students over multiple years in the intervention, which will help combat the development of obesity over time. Examining the PA trends and dietary patterns of these children in future studies would also support the effectiveness of the intervention.

## Conclusion

The results of this pilot study added evidence to existing research showing a significant difference in how BMI and BIA classify students into different body composition classifications. This seems to be especially evident in populations with a high amount of PA, in which BIA may be a more accurate assessment of obesity than BMI. Future researchers should use caution when using BMI to assess obesity rates among children and should seek alternative methods such as BIA, especially when the data is assessed in a place other than a lab setting. BIA has been found to be a valid and reliable assessment tool that can be used specifically in a field setting. Therefore, as shown in multiple studies, BIA takes us a step closer to a field based assessment that can be used in large children populations with consistency. Further analysis is needed to determine if hydration and time of day creates a larger gap in category identification than this study reported.

## Data Availability Statement

The raw data supporting the conclusions of this article will be made available by the authors, without undue reservation.

## Ethics Statement

The studies involving human participants were reviewed and approved by Texas Christian University. Written informed consent to participate in this study was provided by the participants' legal guardian/next of kin.

## Author Contributions

DF wrote a significant portion of the manuscript, collected data, and completed statistical analysis. DR director of the LiiNK Project who assisted in setting up data collection at the schools, writing of the manuscript, and providing edits/revisions. All authors contributed to the article and approved the submitted version.

## Funding

This research was funded by the Paul E. Andrews Foundation.

## Conflict of Interest

The authors declare that the research was conducted in the absence of any commercial or financial relationships that could be construed as a potential conflict of interest.

## Publisher's Note

All claims expressed in this article are solely those of the authors and do not necessarily represent those of their affiliated organizations, or those of the publisher, the editors and the reviewers. Any product that may be evaluated in this article, or claim that may be made by its manufacturer, is not guaranteed or endorsed by the publisher.

## Abbreviations

BIA: Bioelectrical impedance analysis
PA: Physical activity
BF: Body fat
BMI: Body mass index
MVPA: Moderate to vigorous physical activity
PE: Physical education.
